# A Novel Aldosterone Antagonist Limits Renal Injury in 5/6 Nephrectomy

**DOI:** 10.1038/s41598-017-08383-2

**Published:** 2017-08-11

**Authors:** Clarice K. Fujihara, M. C. Kowala, M. D. Breyer, Claudia R. Sena, Mariliza V. Rodrigues, Simone C. A. Arias, Camilla Fanelli, Denise M. Malheiros, P. K. Jadhav, Chahrzad Montrose-Rafizadeh, Jose E. Krieger, Roberto Zatz

**Affiliations:** 1 Faculty of Medicine, University of São Paulo, São Paulo, Brazil; 2 Lilly Research Laboratories, Indianapolis, IN USA

## Abstract

Aldosterone antagonists slow the progression of chronic kidney disease (CKD), but their use is limited by hyperkalemia, especially when associated with RAS inhibitors. We examined the renoprotective effects of Ly, a novel non-steroidal mineralocorticoid receptor (MR) blocker, through two experimental protocols: In Protocol 1, male Munich-Wistar rats underwent 5/6 renal ablation (Nx), being divided into: Nx+V, receiving vehicle, Nx+Eple, given eplerenone, 150 mg/kg/day, and Nx+Ly, given Ly, 20 mg/kg/day. A group of untreated sham-operated rats was also studied. Ly markedly raised plasma renin activity (PRA) and aldosterone, and exerted more effective anti-albuminuric and renoprotective action than eplerenone. In Protocol 2, Nx rats remained untreated until Day 60, when they were divided into: Nx+V receiving vehicle; Nx+L treated with losartan, 50 mg/kg/day; Nx+L+Eple, given losartan and eplerenone, and Nx+L+Ly, given losartan and Ly. Treatments lasted for 90 days. As an add-on to losartan, Ly normalized blood pressure and albuminuria, and prevented CKD progression more effectively than eplerenone. This effect was associated with strong stimulation of PRA and aldosterone. Despite exhibiting higher affinity for the MR than either eplerenone or spironolactone, Ly caused no hyperkalemia. Ly may become a novel asset in the effort to detain the progression of CKD.

## Introduction

In addition to its mineralocorticoid effects, aldosterone (Aldo) is a major contributor to renal fibrosis in chronic kidney disease (CKD)^1, 2^. The mechanisms underlying the profibrotic effects of Aldo are obscure. However, the well-known observation that mineralocorticoid receptor (MR) antagonists, such as spironolactone (Spiro) and eplerenone, (Eple) exert a renoprotective effect in CKD^3–6^ indicate that binding to the MR is as crucial to the inflammatory effect of Aldo as to its effect on sodium and potassium excretion.

A major untoward effect of MR blockers is hyperkalemia, especially in association with inhibitors of the renin-angiotensin system (RAS). Therefore, the development of Aldo antagonists with sizable antifibrotic properties and little effect on potassium is highly desirable.

In recent years, a third generation of MR blockers, possessing a nonsteroidal structure, has emerged^7–9^. These compounds are more selective than Spiro or Eple, exhibiting much higher affinity for the MR than for the glucocorticoid (GR), androgen (AR) or progesterone (PR) receptors. In addition, they appear to promote less hyperkalemia than their predecessors^10^. Recent clinical and experimental observations suggest that these compounds may be used to slow the progression of CKD^9–11^.

Ly is a novel nonsteroidal compound that has been shown, in preliminary experiments, to bind selectively and with very high affinity to the human MR. In the present study, we investigated whether Ly, in monotherapy or as an add-on to the AT-1 receptor blocker, losartan (L), can slow the progression of CKD in rats with 5/6 renal ablation.

## Results

### Molecular structure of Ly

**Figure 1:**
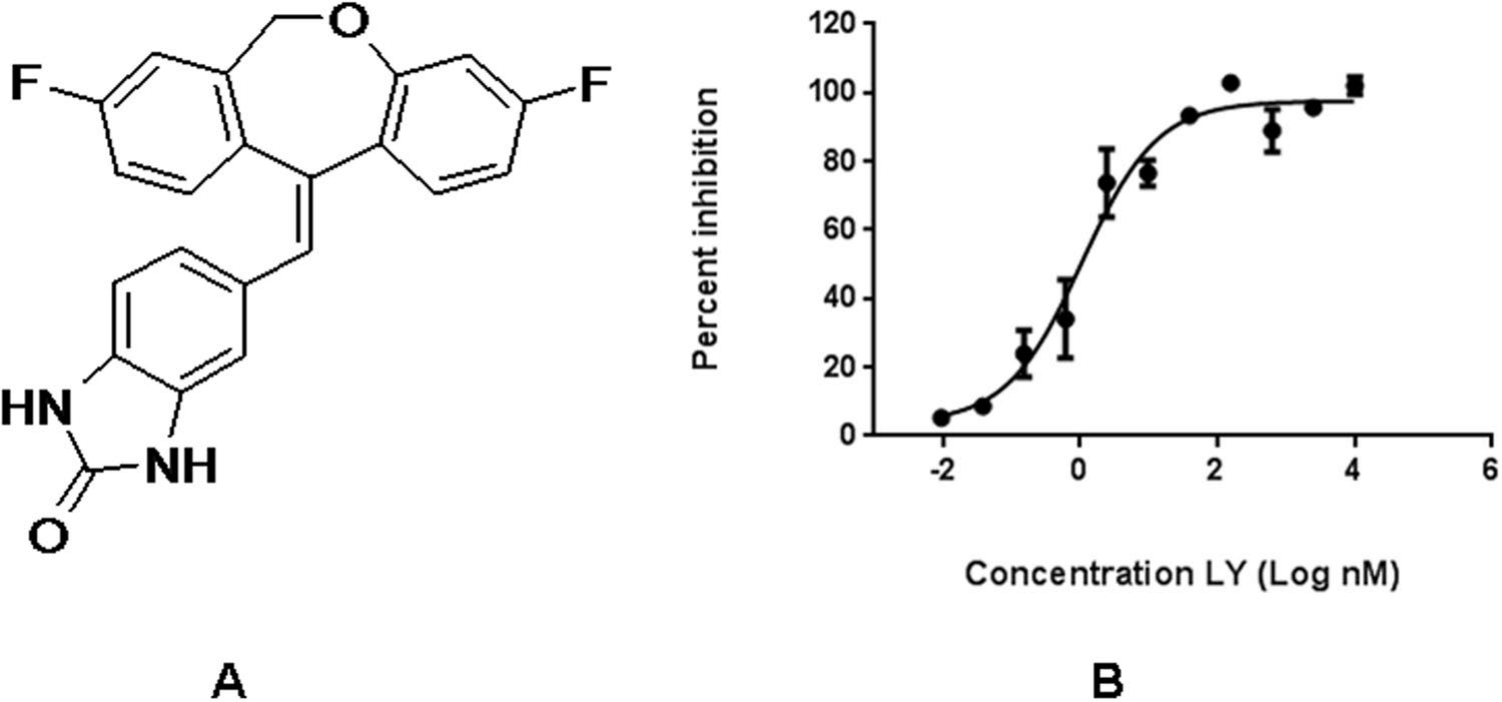
(**A**) The molecular structure of Ly. (**B**) Concentration-response curve of the MR antagonist Ly against human MR (n = 4 separate experiments).

Ly is a non-steroidal MR antagonist with a molecular weight less than 400 Daltons (Fig. 1A). Ly is built on a dibenzooxepine chemical platform with no chiral centers and the key hydrogen bond donor is contributed by the benzimidazolone substructure. Ly has good permeability and good pharmacokinetics properties (data not shown) which contributes to good *in vivo* activity and it contains chemically stable functional groups which are needed for improving binding to MR. Another third generation non-steriodal MR antagonist, Finerenone, has a dihydronaphthyridine scaffold, and a chiral center with S absolute configuration. The key hydrogen bond donor in Finerenone is contributed by the dihydronaphthyridine substructure.

### Binding studies

The binding inhibition curve for Ly against MR is shown in Fig. 1B. Results of the binding studies are shown in Table 1. The K_i_ of Spiro for the MR was 2.4 nM, whereas Eple exhibited a K_i_ two orders of magnitude higher, indicating much lower affinity for the MR. The K_i_ of Ly was 1.6, comparable to that of Spiro. Although the PR:MR K_i_ ratio for Spiro was 167, the AR:MR and GR:MR K_i_ ratios were only 16 and 14, respectively, indicating the relatively low selectivity of this compound. Eple exhibited much higher MR selectivity, with AR:MR and PR:MR K_i_ ratios of >73 and 234, respectively, but with a GR:MR K_i_ ratio of only 8. As for Ly, the AR:MR, PR:MR and GR:MR K_i_ ratios were 202, 58 and 70, respectively, showing that this compound was more uniformly selective for the MR than either Spiro or Eple.

**Table 1:** Binding K_i_ for Nuclear Receptors expressed in Sf9 and HEK293 Cells.

Mean K_i_ ± SEM (nM)					Ratio of K_i_’s		
	**hMR** (**nM**)	**hAR** (**nM**)	**hPR** (**nM**)	hGR* (**nM**)	**hAR: hMR**	**hPR: hMR**	hGR*: hMR
Spironolactone	2.4 ± 0.2 (n = 34)	39 ± 9 (n = 14)	400 ± 83 (n = 16)	33 ± 7 (n = 5)	16	167	14
Eplerenone	124 ± 21 (n = 27)	>9000 (n = 2)	29 K ± 16 K (n = 8)	924 ± 99 (n = 2)	>73	234	8
**Ly**	1.6 ± 0.3 (n = 5)	323 ± 101 (n = 4)	92 ± 19 (n = 4)	112 ± 18 (n = 3)	202	58	70

Mean ± 1 SE; hMR human mineralocorticoid receptor; hAR human androgen receptor; hPR human progesterone receptor; hGR* human glucocorticoid receptor.

### Protocol 1

**Figure 2:**
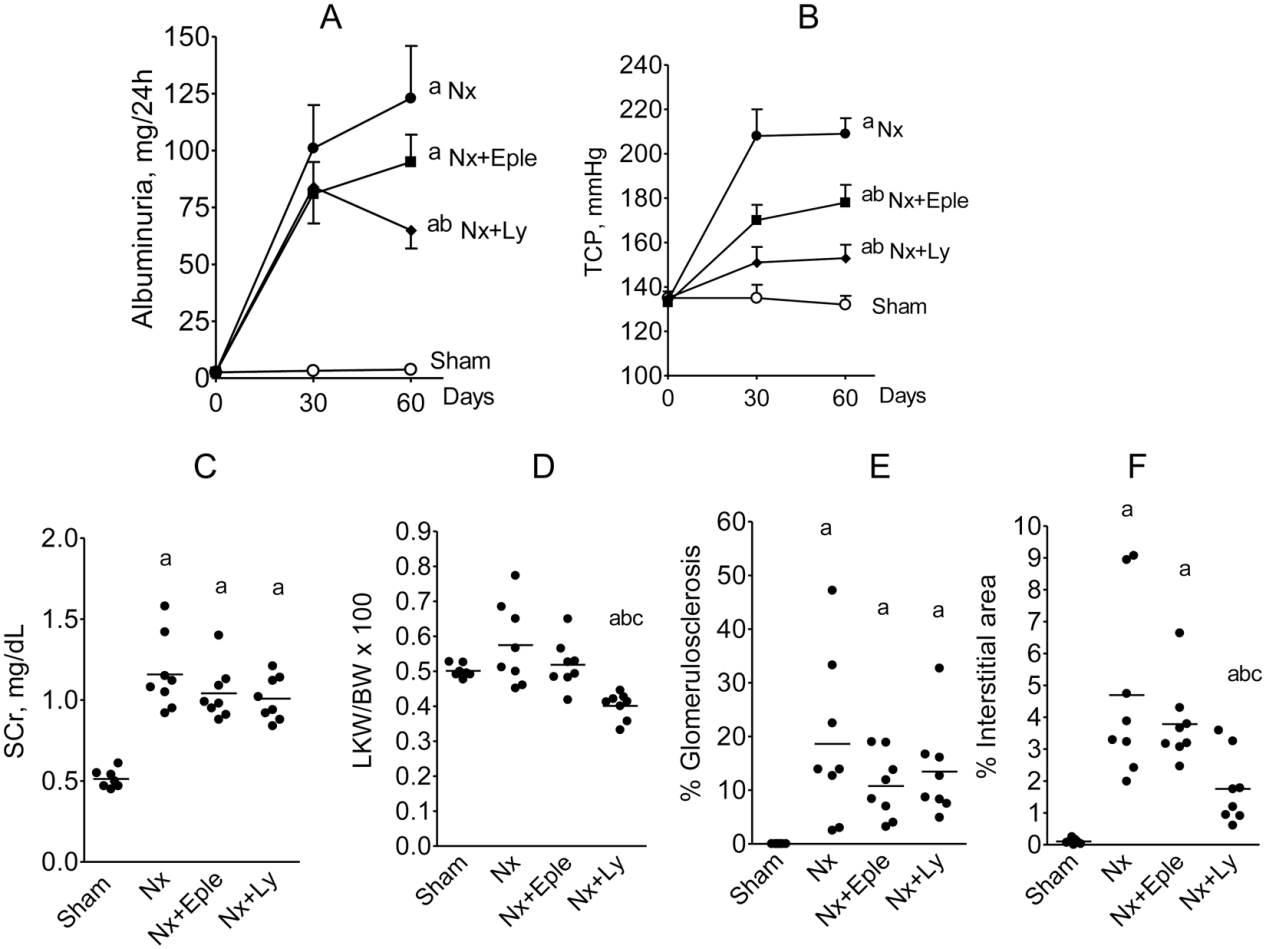
Albumin excretion rate (**A**), tail-cuff pressure (**B**), serum creatinine (**C**), left kidney weight corrected for body weight (**D**), fraction of glomeruli with sclerotic lesions (**E**) and fractional cortical interstitial area (**F**) 60 days after renal ablation in Protocol 1 (early treatment). ^a^p < 0.05 vs. Sham, ^b^p < 0.05 vs. Nx, ^c^p < 0.05 vs. Nx+Eple.

Body growth was limited in all Nx groups compared to S, with no difference among Nx groups (data not shown). As shown previously, an intense and progressive increase in albumin excretion rate (U_alb_V) was observed in untreated Nx rats (Fig. 2A), which was slightly, but not significantly, attenuated by Eple 60 days after ablation. Albuminuria was significantly lower in Ly-treated than in untreated or Eple-treated rats, though still abnormally high. Tail-cuff pressure (TCP) (Fig. 2B) was markedly and persistently elevated in Group Nx + V. Eple treatment partially prevented the elevation of TCP. More pronounced attenuation of hypertension was obtained with Ly, although a statistically significant difference with Group Nx + Eple was not achieved. Serum creatinine (Fig. 2C) was significantly increased in all Nx groups, without differences among them.

Kidney weight was numerically higher in Group Nx + V compared to S, indicating marked enlargement of the remnant kidney. Eple had no effect on renal weight, whereas renal hypertrophy was significantly attenuated in Ly-treated rats (Fig. 2D).

**Figure 3:**
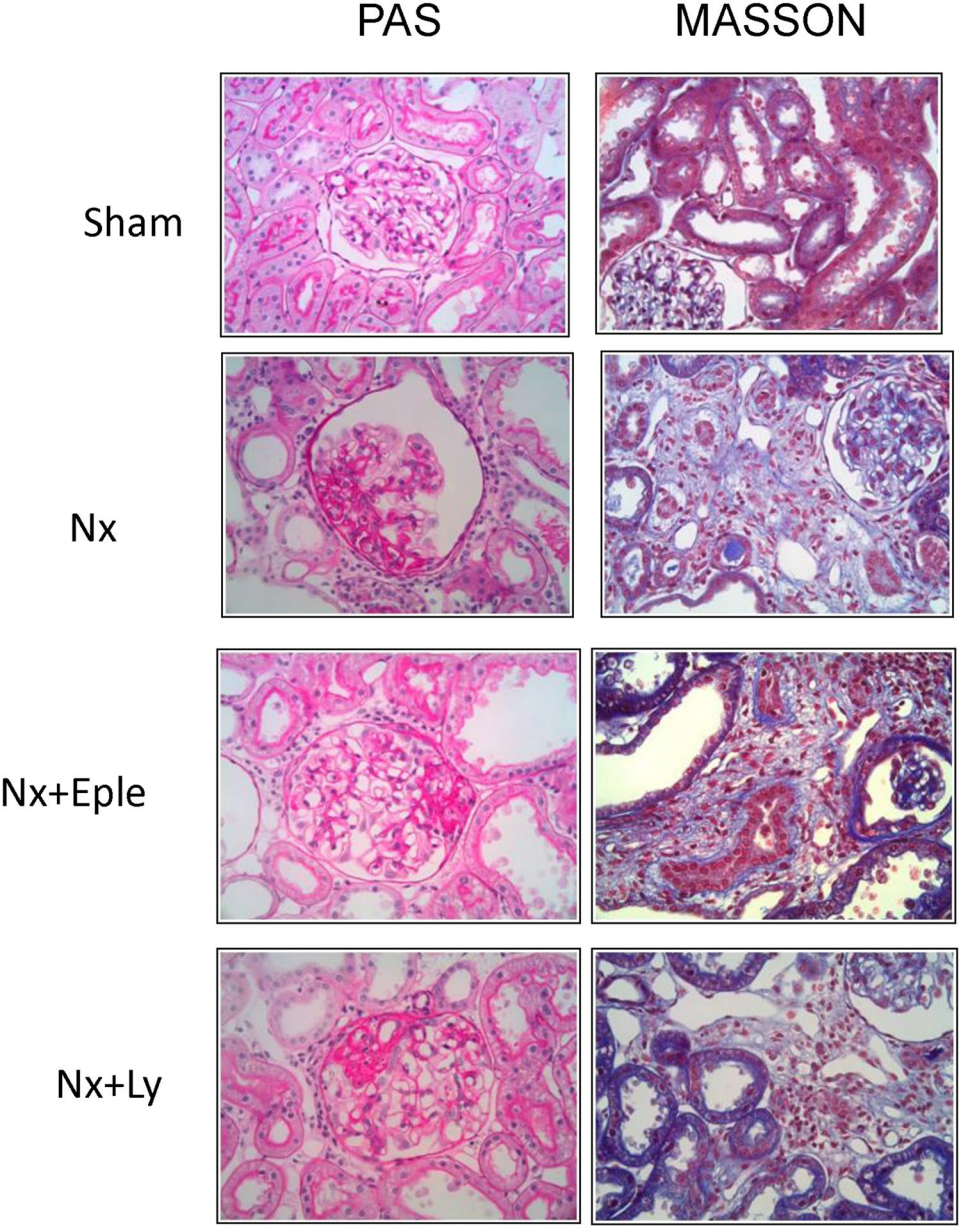
Representative photomicrographs (x400) of PAS-stained (for glomerulosclerosis) and Masson-stained (for cortical interstitium) renal tissue in the four groups studied in Protocol 1 (early treatment).

Untreated Nx rats exhibited widespread glomerulosclerosclerosis (Fig. 2E), which was partially prevented by both treatments. Fractional interstitial area was markedly increased in Nx rats, only slightly attenuated by Eple, and significantly diminished by Ly treatment (Fig. 2F). Representative microphotographs of PAS-stained (for GS) and Masson-stained (for interstitium) renal tissue are shown in Fig. 3.

**Figure 4:**
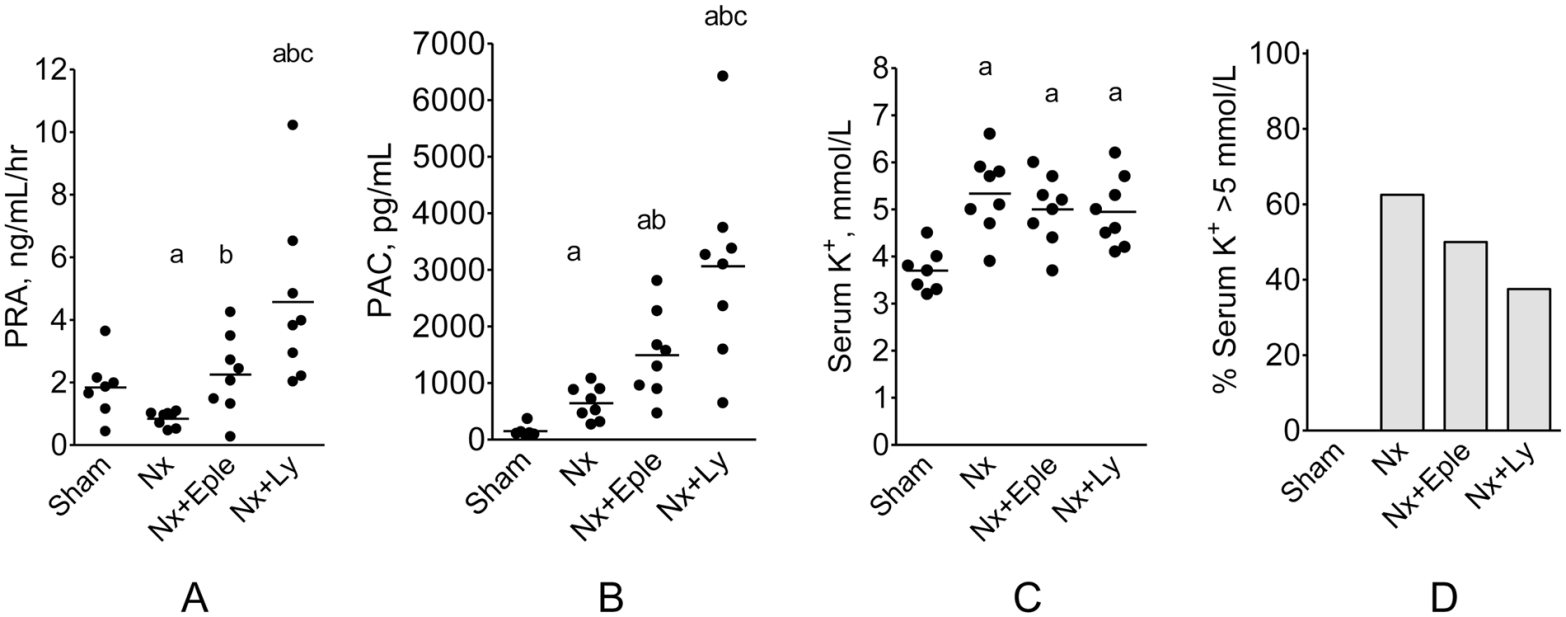
Plasma renin activity (PRA, (**A**), Plasma Aldosterone Concentration (PAC, (**B**), serum K^+^ (**C**) and percent hyperkalemia, defined as serum K^+^ > 5 mmol/L (**D**) 60 days after renal ablation in Protocol 1 (early treatment). ^a^p < 0.05 vs. Sham, ^b^p < 0.05 vs. Nx, ^c^p < 0.05 vs. Nx+Eple.

Plasma renin activity (PRA) was numerically lower in untreated Nx rats, and similar to control in Eple-treated rats, whereas markedly increased PRA was observed in Ly-treated rats (Fig. 4A). Plasma aldosterone concentration (PAC) was moderately increased in Nx rats (Fig. 4B). Treatment with Eple exacerbated hyperaldosteronemia, whereas Ly raised PAC to levels over 30-fold higher than in S.

A significant increase in plasma K^+^ levels was observed in Group Nx + V, which was not aggravated by either Eple or Ly treatment (Fig. 4C). The same profile was observed when hyperkalemia (defined as plasma K^+^ > 5) was expressed as a categorical variable (Fig. 4D).

### Protocol 2

**Figure 5:**
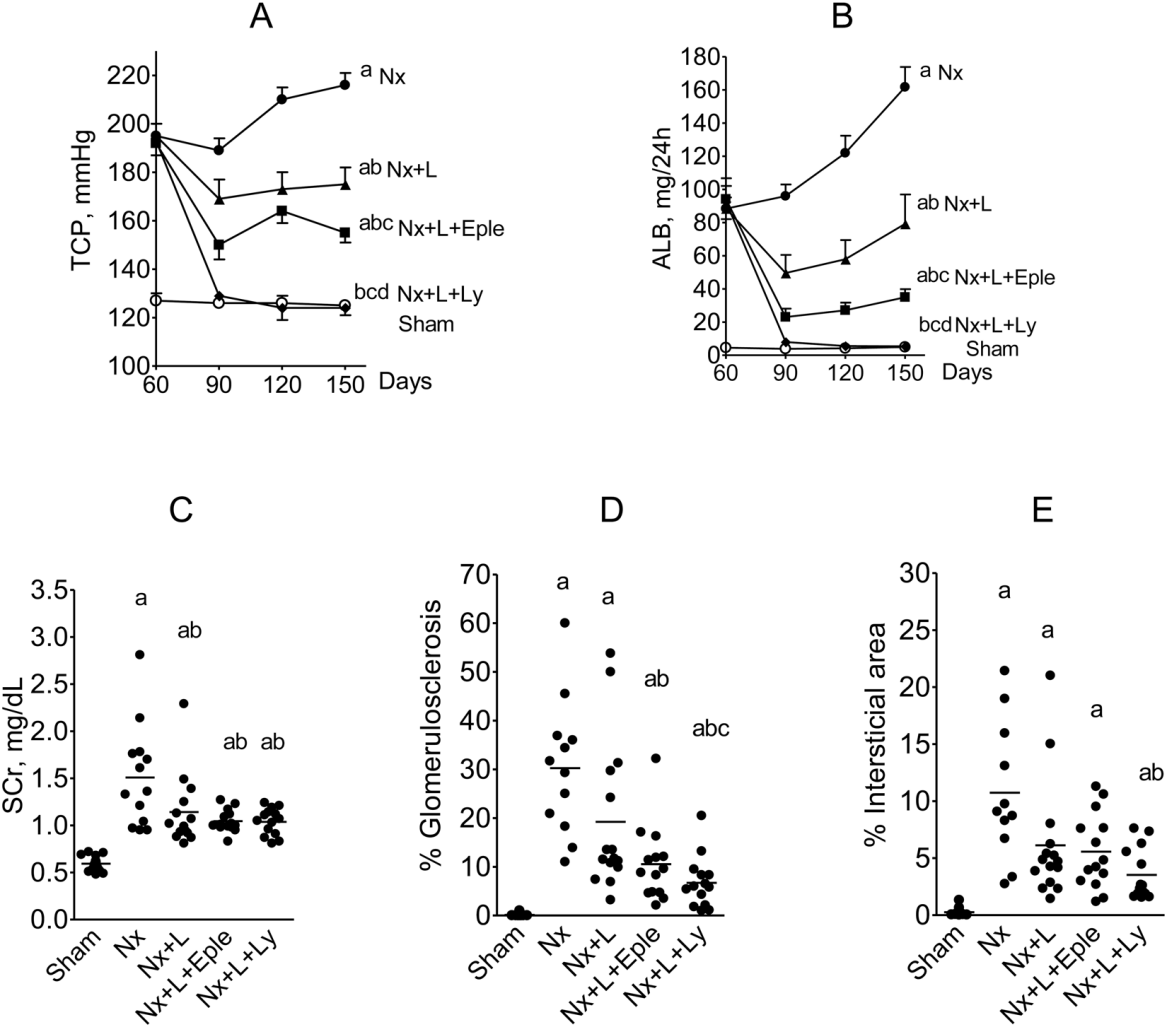
Tail-cuff pressure (**A**), albumin excretion rate (**B**), serum creatinine (**C**), percentage of glomeruli with sclerotic lesions (**D**) and fractional cortical interstitial area (**E**) 150 days after renal ablation in Protocol 2 (late treatment). ^a^p < 0.05 vs. Sham, ^b^p < 0.05 vs. Nx, ^c^p < 0.05 vs. Nx+Eple.

All Nx groups exhibited slower growth compared to S, with no difference among them (not shown). In untreated Nx rats, TCP was severely and progressively elevated (Fig. 5A), exceeding 210 mmHg 150 days after renal ablation. Hypertension was mitigated by L monotherapy and by the L + Eple association. L + Ly treatment promoted sustained normalization of TCP. Likewise, massive and progressive albuminuria was observed in Nx + V rats (Fig. 5B). L treatment prevented the progression of albuminuria. L + Eple treatment significantly lowered U_alb_V. With the L + Ly scheme, U_alb_V remained close to control until the end of the study. As in Protocol 1, serum creatinine (Fig. 5C) was significantly increased by the ablation procedure, with no statistical differences between Nx groups.

**Figure 6:**
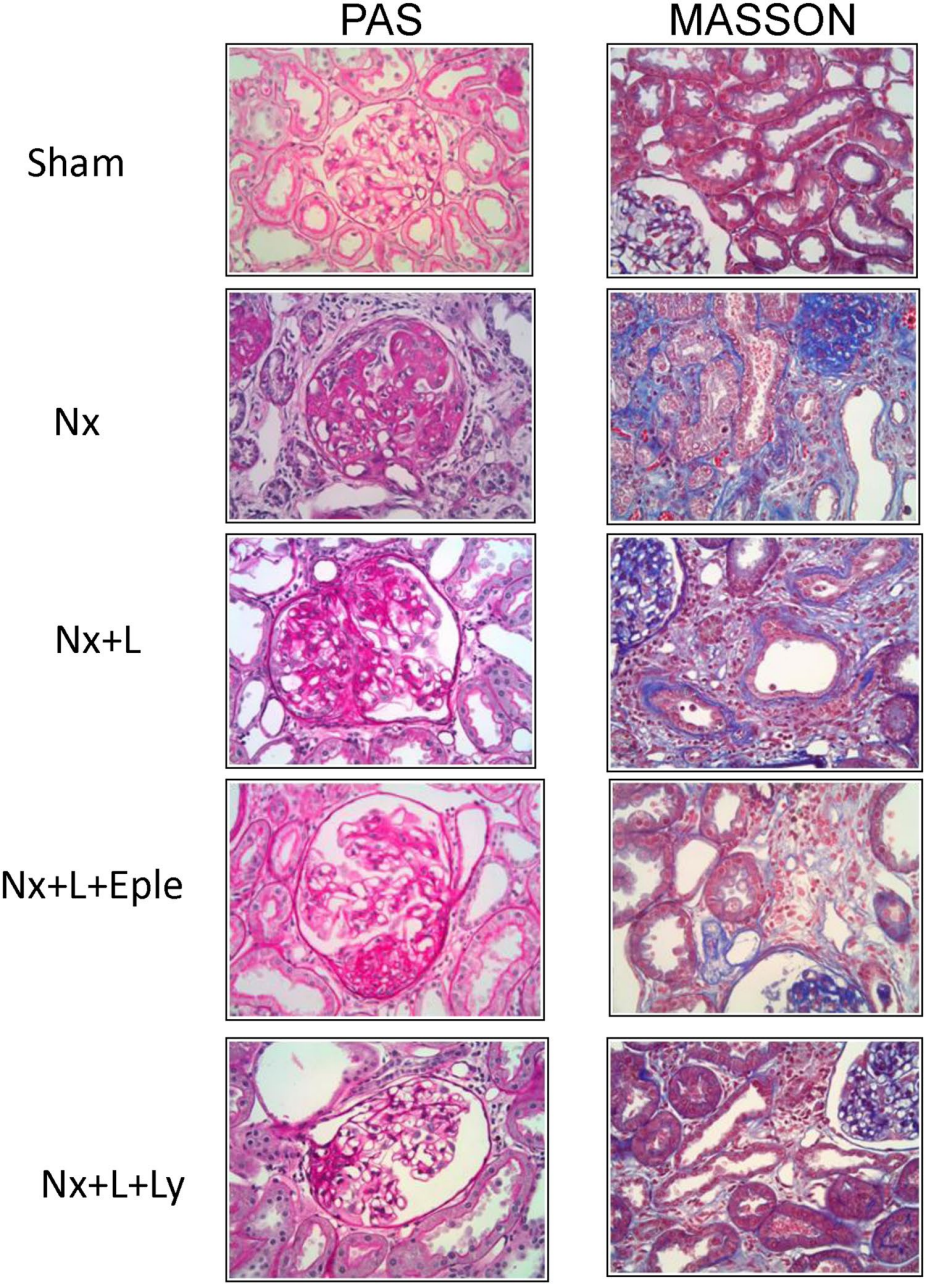
Representative photomicrographs of PAS-stained (for glomerulosclerosis) and Masson-stained (for cortical interstitium) renal tissue in the five groups studied in Protocol 2 (x400).

**Figure 7:**
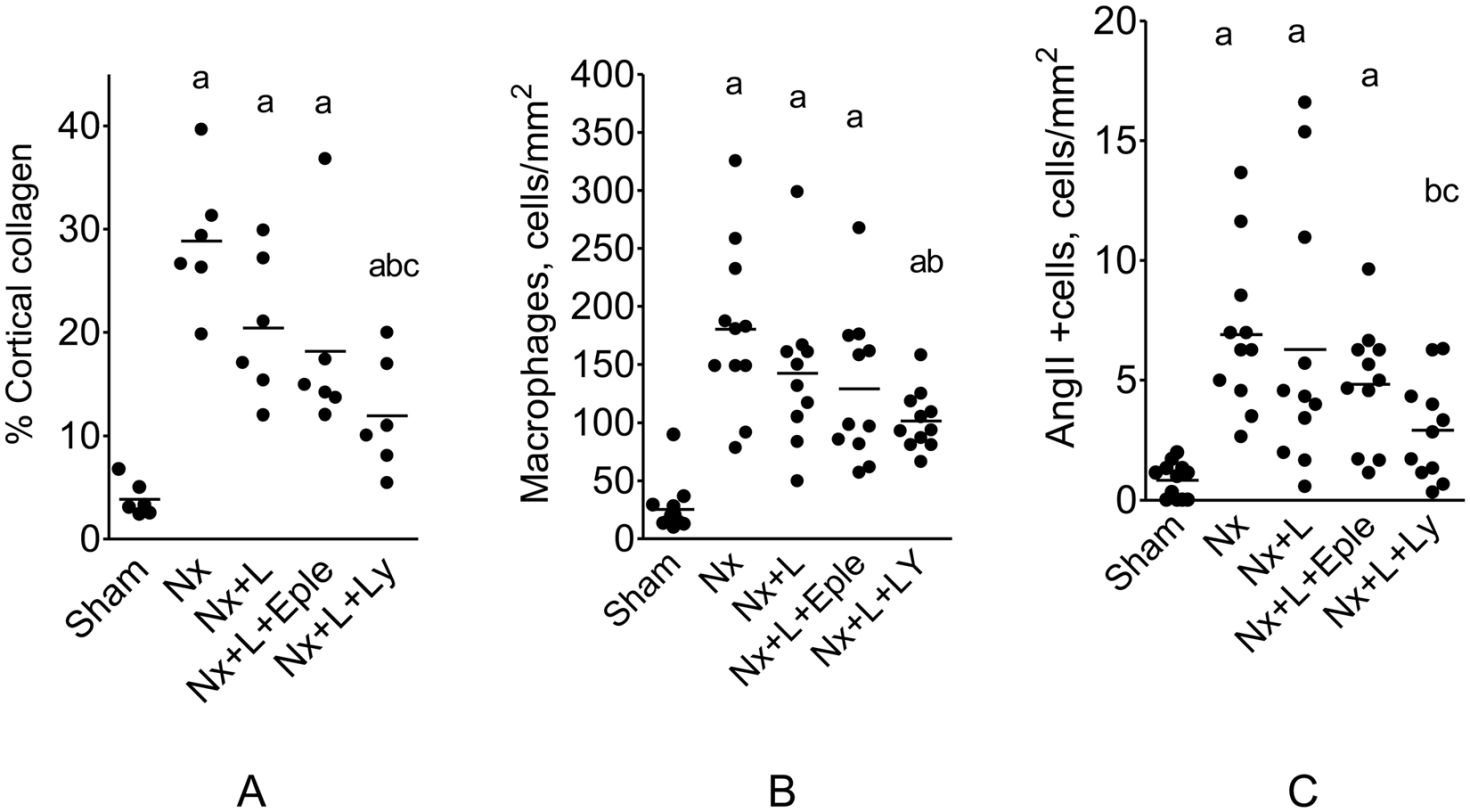
Percent cortical area occupied by collagen I (**A**) and the density of interstitial infiltration by macrophages (**B**) and AngII+ cells (**C**) 150 days after renal ablation in Protocol 2 (late treatment). ^a^p < 0.05 vs. Sham, ^b^p < 0.05 vs. Nx, ^c^p < 0.05 vs. Nx+Eple.

The fraction of sclerotic glomeruli was markedly increased in untreated Nx rats compared to S (Fig. 5D). L monotherapy partly prevented GS, while L + Eple treatment reduced GS% more effectively. Renoprotection was maximal with the L + Ly association, which lowerd %GS compared to Nx and Nx + L. Parallel results were obtained for the cortical interstitial area (Fig. 5E). Representative microphotographs of PAS-stained (for GS) and Masson-stained (for interstitium) renal tissue in Protocol 2 are shown in Fig. 6. The cortical area occupied by collagen I was markedly expanded in untreated Nx (Fig. 7A). Both L monotherapy and the L + Eple regimen failed to reduce significantly the extent of interstitial fibrosis, which was achived only with the L + Ly treatment.

The intensity of interstitial macrophage infiltration (Fig. 7B), which was markedly elevated in Group Nx + V, was numerically but not significantly reduced in Groups Nx + L and Nx + L + Eple. The L + Ly treatment brought the macrophage infiltration to levels significantly lower than in Nx + V, though still higher than in S.

**Figure 8:**
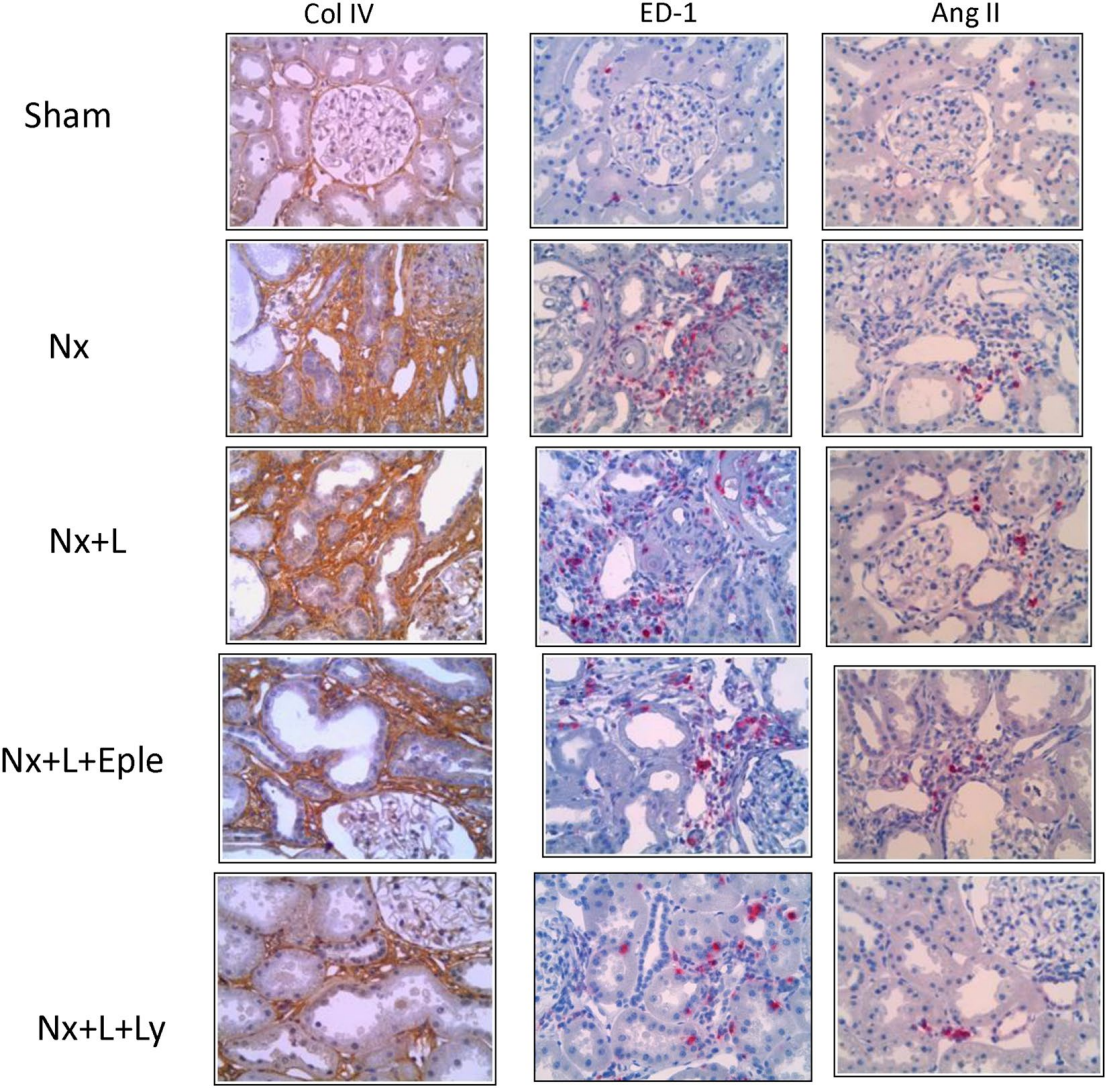
Representative photomicrographs of renal tissue stained by immunohistochemistry for collagen I, macrophages and AngII-+ cells in the five groups studied in Protocol 2 (x400).

AngI+interstitial cells (Fig. 7C), infrequently observed in S, abounded in the Nx + V group. The density of AngII+cells was not significantly altered by either L or L + Eple treatment. The L + Ly association brought AngII + infiltration to levels significantly lower than in Nx + V, and comparable to the S value. Figure 8 shows representative photomicrographs of renal tissue stained by immunohistochemistry for collagen I, macrophages and AngII-positive cells in Protocol 2.

### Effect of Nx and treatments on PRA, PAC and serum K+

**Figure 9:**
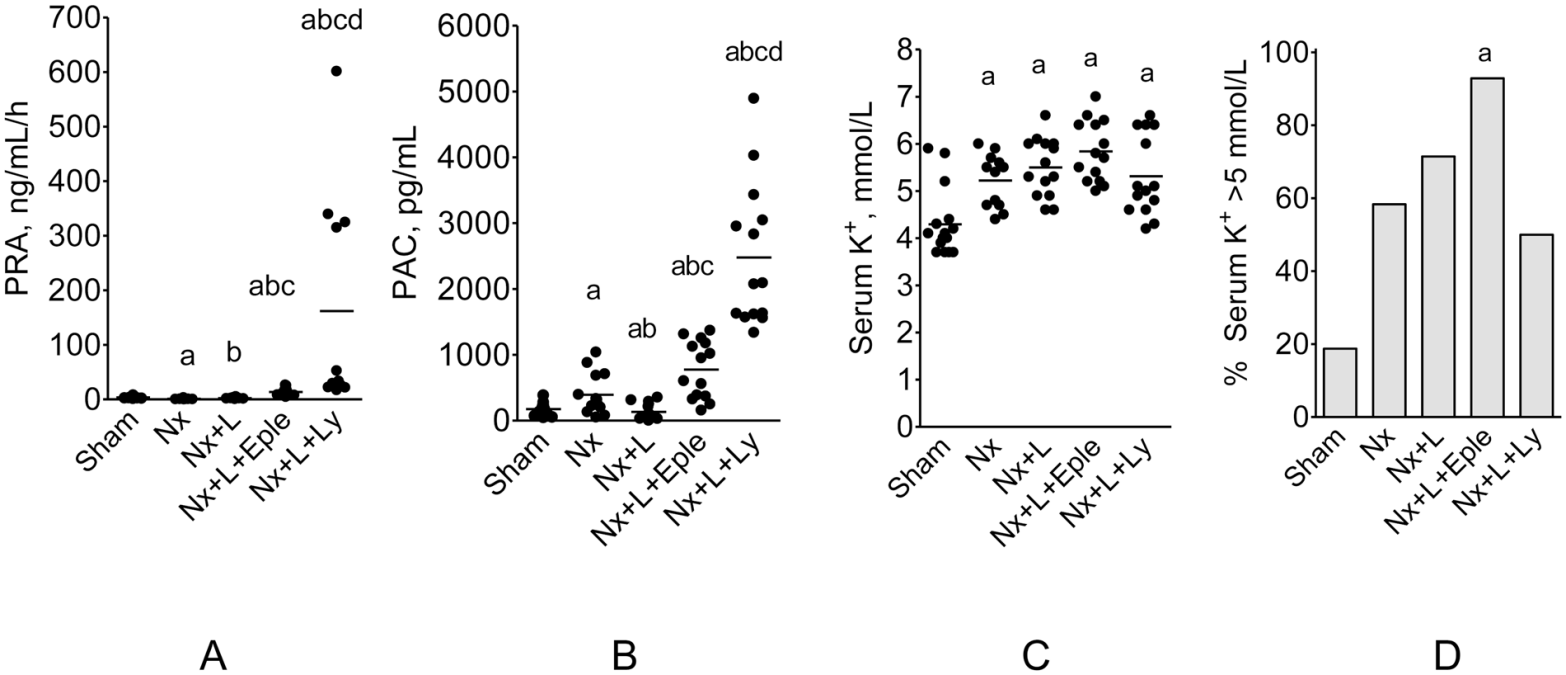
Plasma renin activity (PRA) (**A**), plasma aldosterone concentration (PAC) (**B**), serum K^+^ (**C**) and percent hyperkalemia, defined as serum K^+^ > 5 mmol/L (**D**) 150 days after renal ablation in Protocol 2. ^a^p < 0.05 vs. Sham, ^b^p < 0.05 vs. Nx, ^c^p < 0.05 vs. Nx+Eple.

In untreated Nx, PRA was significantly decreased compared to S (Fig. 9A). L monotherapy normalized PRA. In Group Nx + Eple, PRA was significantly elevated compared to S, Nx and Nx + L. This effect was much stronger in the Nx + L + Ly group, in which PRA was over 100 fold as high as in S. In Nx rats, PAC was significantly elevated relative to control (Fig. 9B). L treatment significantly reduced PAC compared to S or Nx + V. The L + Eple regimen markedly raised PAC compared to Groups S, Nx + V and Nx + L. PAC was maximal in Group Nx + L + Ly, reaching over 30 fold the S value.

As in Protocol 1, serum K^+^ (Fig. 9C) was similarly elevated in all Nx groups. Surprisingly, K^+^ values were numerically lower in the Nx + L + Ly group compared to the other Nx groups. A similar pattern was observed when hyperkalemia (serum K^+^ > 5 mmol/L) was expressed as a categorical variable (Fig. 9D), although only the difference between Group Nx + L + E and Sham achieved statistical significance.

## Discussion

Our binding experiments corroborated previous findings that, though exhibiting much lower affinity than Spiro for the MR, Eple is much more selective regarding the AR, but not the PR or GR. On the other hand, the affinity of the Ly compound for the MR is comparable to that of Spiro, while its selectivity relative to the AR, PR and GR is much higher than that of either Spiro or Eple.

As expected, PRA was depressed in Nx + V rats, reflecting the volume expansion associated with this model. The increase in PRA observed with Eple monotherapy indicates some degree of volume loss. The elevation of PAC that paralleled Eple treatment may have resulted directly from hyperreninemia, also consistent with volume contraction. These effects on PRA and PAC were much more intense with Ly monotherapy, suggesting that the higher MR affinity of this compound may have promoted more intense volume depletion. Consistent with this view is the fact that Ly acted as a better antihypertensive than Eple. This hemodynamic effect may also underlie, at least in part, the renoprotective effect of Ly.

Late-onset L monotherapy limited hypertension and albuminuria compared to the N + V group, without significant reduction of renal injury or inflammation. These results confirm previous observations of this laboratory^12–14^, and are analogous to those obtained in clinical studies^15–17^, in which RAS suppressors have been shown to retard, but not suppress, CKD progression.

The antihypertensive and antiproteinuric effects of L were enhanced when combined with Eple, an expected finding in view of the complementarity of their mechanisms of action^3, 18, 19^, although macrophage infiltration, GS and collagen accumulation were not significantly altered in comparison with Nx + L. These observations are in agreement with previous reports that, in the Nx model, these combined treatments, despite favorable hemodynamic effects, do not necessarily exert more effective renoprotection than ARBs or ACE inhibitors alone^18, 19^.

The salutary effects of Ly as an add-on to L were clearly superior to those of Eple: besides normalization of TCP and U_alb_V, GS, interstitial expansion and collagen deposition were reduced to levels significantly lower than in the Nx + V group, though still higher than in S. Likewise, Ly add-on treatment exerted a clear anti-inflammatory effect, limiting renal interstitial infiltration by macrophages and AngII+ cells.

The effects of add-on treatment with Eple, and especially Ly, on the RAS and PAC are worth of consideration. As with monotherapies, both MR blockers raised PRA and PAC, but the increases promoted by Ly were much more intense than with Eple. The mechanism of this effect of simultaneous AT-1 and MR blockade on PAC, reported previously in clinical and experimental studies^20–22^, is unclear. In the present study, the significant linear correlation observed between PRA and PAC suggests volume depletion as the main stimulus for increased Aldo production. However, Aldo production must have been independent of AngII, since in Protocol 2 the AT-1 receptor was blocked by L. One obvious alternative stimulus could be serum K^+^, which exerts a well-known direct effect on the zona glomerulosa^23, 24^. However, no significant differences in serum K^+^ were observed. Actually, mean serum K^+^ and the frequency of values in excess of 5 mmol/L were numerically lower in Nx + L + Ly than in Nx + L + Eple, even though the increase in PAC was much greater in the former, suggesting that other factors may have governed Aldo production.

Of particular interest is the dissociation between PRA and the presence of AngII+ cells: whereas the profile of PRA among groups suggested a coherent response to volume depletion, the intensity of interstitial infiltration by AngII+ cells followed a completely opposite trend, underlining the inflammatory nature of locally produced AngII^25^.

The reasons why Ly exerted better renoprotection than Eple as an add-on to L are unclear: Conceivably, the superiority of Ly might simply reflect the use of a submaximal dose of Eple. However, this is an unlikely possibility, since both drugs were used at their maximal nontoxic doses, defined by stunting of body growth. It should be stressed that the doses of Ly (20 mg/kg) and Eple (150 mg/kg) employed in this study were not equipotent: whereas the Eple/Ly K_i_ ratio (124/1.6) was 77.5, indicating that Ly exerted a much more effective MR blockade, the respective molar dose ratio was only 7.1. This higher affinity of Ly for the MR may have resulted in less activation of profibrotic intracellular pathways. However, such explanation is not easily reconciled with the fact that Ly promoted no more hyperkalemia than Eple (in fact, hyperkalemia was less frequent in Ly- than in Eple-treated rats, although the difference between the two groups did not achieve statistical significance), an unexpected finding given that major downstream mediators, such as SGK1, drive both proinflammatory and mineralocorticoid effects of Aldo. On the other hand, the very high aldosterone levels associated with Ly treatment may have promoted a “breakthrough” effect^26^, perhaps through a differential effect on nongenomic pathways, although such hypothesis is at present merely speculative.

Another explanation for the better renoprotection afforded by the L + Ly scheme could be its better antihypertensive effect. This hypothesis is rather difficult to test, since better renal preservation inevitably results in more effective antihypertensive action, given the key role of the kidneys on blood pressure^27^. However, previous studies of experimental CKD showed that the renoprotective and antihypertensive actions of MR blockers can be dissociated^5, 28^. Moreover, we showed recently that normalization of blood pressure in Nx with a triple therapy was unable to reproduce the renoprotective action of a combined L/thiazide scheme^12^. Lastly, a more vigorous volume depletion by L + Ly compared to L + Eple, suggested by the observed effects on PRA, may have mimicked the beneficial effect exerted by diuretics when combined with ACEI or ARBs^12, 13, 29^.

To the best of our knowledge, this is the first time a renoprotective effect of a third generation, nonsteroidal MR blocker is reported in the Nx model. The implications of these findings, should Ly and similar compounds prove equally effective and nontoxic in other experimental CKD models and in clinical studies, are self-evident and may result in the incorporation of a valuable novel asset to the arsenal available for the management of CKD.

In summary, we showed that Ly, a third-generation MR blocker, is superior to Eple as an antihypertensive, antiproteinuric and renoprotective agent in the Nx model, without aggravating hyperkalemia. The exact mechanisms underlying the greater efficacy of Ly over eplerenone are unclear, but could relate to a stronger MR blockade. Future studies will define whether similar protection can be obtained in other models of CKD, and whether these findings can be safely translated to clinical practice.

## Methods

### Binding studies

Full-length human mineralocorticoid receptor (hMR) to be used in radioligand binding was isolated from a human kidney cDNA library (Clontech, Palo Alto, CA). The human progesterone receptor (hPR) cDNA sequence was almost identical to the Genebank entry M15716 except for a 21 base pair (bp) deletion in the amino terminus. The human androgen receptor (hAR) cDNA was sequenced and was almost identical to the Genebank entry M20132, except at nucleotide (NT) position 388 where an A/G mutation resulted in a substitution of G for R and the inclusion of a 27-repeat polygly-polymorphism (instead of 24) in the N-terminal domain. The hMR, hPR, and hAR cDNAs were subcloned in the eukaryotic expression vector pcDNA3. Methods for the expression of functionally active hMR, hAR and hPR utilizing the baculovirus expression system were described previously^30, 31^.

Affinity of ligands to the soluble receptor was determined using vacuum filtration methods, preparing soluble receptors in 96-well Falcon polystyrene plates (BD Biosciences, Bedford, MA) in a total volume of 250 μl. The hMR Sf9 lysate (approximately 20–25 μg protein in 20 μl) was incubated in the absence or presence of competing ligands with 10 μl of [1,2,6,7-3H] Aldosterone (specific activity 61 Ci/mmol, final concentration 0.5 nM; TRK434 Amersham Pharmacia Biotech, Piscataway, NJ) in a binding buffer containing 30 mM sodium molybdate, 30 mM Tris-HCl, 5 mM sodium phosphate, 5 mM sodium pyrophosphate and 10% glycerol, pH 7.45. Binding studies for ligands for hPR and hAR were determined using [1,2,6,7-3H(N)] Progesterone (specific activity 103 Ci/mmol, at a final concentration 0.5 nM; NET381, NEN/Perkin Elmer Life Sciences, Boston, MA) and [17α-methyl-3H] Mibolerone, an enzymaticaly stable analog of testosterone (specific activity 76.8 Ci/mmol, final concentration 0.3 nM; NET919, NEN), respectively. Specific binding of 3H-progesterone for hPR and 3H-mibolerone for hAR was determined in the presence and absence of 1 μM mifepristone. Inhibition constants (Ki), were determined either from the Cheng-Prusoff equation^32^, or directly using non-linear regression curve fitting to a three-parameter one-site competitive equation. Ki values obtained from individual concentration response curves were reported as the mean ± SEM. Protein was measured using colorimetric detection and quantification of bicinchoninic acid (BCA Protein Assay, Pierce, Rockford, IL). Cos-7L cells were cotransfected with expression vectors and a reporter plasmid responsive to all 3 nuclear hormone receptors^33^. pBluescript II SK (+) (Stratagene, San Diego, CA) was included as a source of carrier DNA. The number of repetitions of full concentration response curves (n) represents values from at least two independent experiments for each condition. Concentration response curves were analyzed statistically using a four-parameter logistic equation (Hill equation for sigmoid curves). Similar methodology was adopted for the human glucocorticoid receptor (hGR) expressed in HEK293 cells.

### Animals

One-hundred forty-five adult male Munich-Wistar rats, obtained from a local facility at the Faculty of Medicine, University of São Paulo, weighing between 220 and 250 g, were used in this study. All experimental procedures were approved by the local Research Ethics Committee (Comissão de Ética para Análise de Projetos de Pesquisa do Hospital das Clínicas da Faculdade de Medicina da Universidade de São Paulo, CAPPesq, process n° 0905/09), and carried out following strictly our institutional guidelines and current international standards for manipulation and care of laboratory animals. Body weights and general conditions were assessed daily. Five-sixths renal ablation (Nx) was performed as a single-step procedure under anesthesia with ketamine 50 mg/kg and xylazine 10 mg/kg im. After ventral laparotomy, the right kidney was excised and two or three branches of the left renal artery were ligated, so as to promote infarction of two-thirds of the left kidney. The operation was invariably well tolerated. Sham-operated rats underwent anesthesia and manipulation of the renal pedicles without removal of renal mass. After surgery, rats received enrofloxacin and, upon recovery, had free access to tap water and regular rodent chow containing 0.5 Na and 22% protein (Nuvital Labs, Curitiba, Brazil), and were kept at 23 ± 1 °C and 60 ± 5% relative air humidity, under an artificial 12–12 hour light/dark cycle. Rats were then treated following two different experimental protocols (described later).

### Determination of the doses of Eple and Ly

Preliminary experiments were performed to exclude the possibility that any differences between Eple and Ly, both in monotherapy and as add-ons to L, were simply due to the use of inadequately low doses of either compound. In the first set of experiments, Nx rats received Eple at the recommended dose of 85 mg/kg, and at 150 mg/kg, higher than in most studies reported so far. No dose-response effect was observed for any of the parameters studied, except for a slight increase in PRA with the 150 mg/kg dose. Use of a higher dose resulted in body growth limitation. Other Nx rats received Ly at either 12 mg/kg (the recommended dose) or 20 mg/kg. A dose-response effect was observed for most parameters. However, administration of a higher dose (30 mg/kg) resulted in severe growth stunting. Based on these preliminary results, we set the doses of Eple and Ly at 150 mg/kg and 20 mg/kg, respectively.

### Early Treatment Protocol (Protocol 1)

In this first protocol, Nx rats were divided into groups: NX + V (N = 8), receiving vehicle; NX + Eple (N = 8), receiving oral (mixed with the chow) Eple, 150 mg/kg/day; and Nx + Ly (N = 8), receiving oral Ly, 20 mg/kg/day. A group of sham-operated rats (S, N = 7) was also studied. Treatments were started 24 h after surgery. Rats were followed during 60 days, with determination of TCP and U_alb_V on Days 30 and 60. TCP was assessed with an optoelectronic automated device (Visitech Systems, Apex, NC), after rats had been preconditioned so as to remain calm during the procedure. U_alb_V was measured with a conventional immunodiffusion technique. On Day 60, rats were anesthetized as described before, and blood was collected from the abdominal aorta for determination of serum creatinine, serum K^+^, PRA and PAC. The remnant kidney was then perfusion-fixed with Duboscq-Brazil solution after a brief washout with saline to remove blood from renal vessels, and prepared for histomorphometric and immunohistochemical analyses using conventional sequential techniques.

### Late Treatment Protocol (Protocol 2)

In Protocol 2, adult male Munich-Wistar rats were subjected to Nx as described earlier, and left untreated for two months. At this time, TCP and U_alb_V were assessed as described earlier. Rats with TCP ≤ 170 mmHg or U_alb_V ≤ 40 mg/day were excluded from the study. The remaining 71 Nx rats were divided into four experimental groups: Nx + V (N = 14), untreated Nx rats; Nx + L (N = 15), receiving oral L, 50 mg/kg in drinking water; Nx + L + Eple (N = 14), receiving L and Eple (N = 14), 150 mg/kg/day; and Nx + L + Ly (N = 14), receiving L and Ly (20 mg/kg/day) as described before. A group of S rats (N = 16) was also included. All groups were followed for 90 days after treatments were started (150 days after nephrectomy), with monthly measurement of TCP and U_alb_V. At the end of this period, rats were anesthetized, blood was collected, and the renal tissue was perfusion-fixed and prepared for histomorphometric and immunohistochemical examination in the same manner as for Protocol 1.

### Histomorphometry

Histomorphometric analysis was performed blindly by two independent observers. The extent of glomerular injury was estimated, in PAS-stained sections, by determining the percentage of glomeruli with sclerotic lesions. The fraction of the renal cortical area occupied by interstitial tissue (%INT) was estimated in 25 consecutive microscopic fields of Masson-stained sections by a point-counting technique^34^, at a final magnification of 200X, under a 144-point grid.

### Immunohistochemical analysis

Immunohistochemistry was performed on 4-μm-thick sections, mounted on glass slides precoated with 2% silane. Sections were deparaffinized and rehydrated by conventional techniques, then heated in citrate buffer for antigen retrieval and incubated overnight with the primary antibody at 4 °C (no antibody in negative control experiments). Primary antibodies used were: monoclonal mouse anti-rat ED-1 (Serotec, Oxford, UK); polyclonal rabbit anti-human AngII (Peninsula Laboratories, San Carlos, CA); and polyclonal rabbit anti-collagen I (Abcam, Cambridge, UK).

For ED-1 detection, sections were preincubated with 5% normal rabbit serum to prevent nonspecific binding, then incubated overnight at 4 °C with the primary antibody diluted in BSA at 1%. After rinsing with Tris-buffered saline (TBS), sections were incubated with an appropriate secondary antibody, then with an alkaline phosphatase anti-alkaline phosphatase (APAAP) complex (Dako, Glostrup, Denmark) and, finally, developed with a fast-red dye solution (Sigma-Aldrich, Saint Louis, MO). For AngII detection, a streptavidin-biotin complex for alkaline phosphatase (Dako, Glostrup, Denmark) was used. Nonspecific binding was prevented with normal horse serum diluted at 1:20 in nonfat milk at 2% in TBS. Primary antibodies were diluted in BSA at 1:200 (for ED-1) or 1:400 (for AngII). Sections were developed in the same manner as for ED-1 detection. For collagen, sections were pretreated with 30% hydrogen peroxide in methanol and preincubated with normal horse serum as described earlier. The primary antibody was diluted at 1:200 in BSA at 1% in TBS. The EnVision Labelled Polymer for peroxydase (Dako, Glostrup, Denmark) was used before development with DAB substrate (Dako, Glostrup, Denmark).

The renal density of macrophages and interstitial AngII+ cells per mm^2^ was blindly evaluated at 400X magnification. For each section, 50 microscopic fields were examined. The percentage of cortical interstitium occupied by collagen I was measured using ImagePro Plus® (Media Cybernetics, Inc., Rockville, MD).

### Plasma renin and Aldo assays

PRA and PAC were determined using commercially available radioimmunoassay kits (Renin Active IRMA and Aldosterone Active RIA, Beckman Coulter, Carlsbad, CA).

### Statistical analyses

Results are Mean ± SE. Differences among groups were assessed using one-way analysis of variance (ANOVA) with pairwise post-test comparisons by the Tukey method^35^. *p* < 0.05 was significant. Calculations and curve fitting were performed using Prism® 4.0 (GraphPad® Software, La Jolla CA). Statistical analysis of the frequency of hyperkalemia (defined as serum K^+^ > 5 mmol/L) was performed using the SPSS™ software, with post test comparisons according to Beasley, T *et al*.^36^.
